# Krukenberg Tumour in a 34-Year-Old Female: A Case Report

**DOI:** 10.7759/cureus.59001

**Published:** 2024-04-25

**Authors:** Atul Chavhan, Prachi Gedekar, Anjali A Vagga, Vishal Ghule, Kaustubh Kharche

**Affiliations:** 1 Pathology, Datta Meghe Institute of Higher Education and Research, Wardha, IND; 2 Medicine, Datta Meghe Institute of Higher Education and Research, Wardha, IND; 3 Biochemistry, Jawaharlal Nehru Medical College, Datta Meghe Institute of Higher Education and Research, Wardha, IND

**Keywords:** chemotherapy, surgery, signet ring cells, adenocarcinoma, epithelial tumor, krukenberg tumor

## Abstract

A metastatic condition involving signet ring cells rich in mucin is the Krukenberg tumor, which affects the ovaries. Usually, the metastatic disease does not include both ovaries and usually originates from the stomach side, while it can also occur less frequently from other locations. Krukenberg tumors are uncommon in younger age groups and usually occur after the age of 40. We report a case of a 34-year-old female patient with a primary form of past medical history. The patient was treated with multiple rounds of chemotherapy.

## Introduction

The term "Krukenberg tumor" refers to ovarian metastatic cancer that typically originates from the gastrointestinal tract and is characterized by mucin-rich signet cell adenocarcinoma and, less commonly, from other sites. Friedrich Krukenberg (1871-1946), a German pathologist and gynecologist, reported what he believed to be a novel form of primary ovarian tumor in 1896. Six years later, it was determined that this lesion was indeed metastatic [1].

According to radiology, Krukenberg tumors are complex, semisolid masses with varying proportions of solid cystic constituents. Solid ovarian secondary lymphomatous involvement usually originates in the upper gastrointestinal tract. However, main colony involvement with cystic disease is more frequent [2].

The average age for diagnosis is 45 years. Abdominal distension, which can occur during pregnancy because of non-pathological components such as fat, fluid, feces, or a fetus is one of the common symptoms of Kugel-Torok syndrome (KT). A physician should also consider other causes such as sepsis, organ failure, and tumors [3]. When diagnosing metastatic ovarian cancers using immunohistochemistry, cytokeratin markers CK20 and CK7 are most frequently used [4].

A review of earlier research indicates that gastric cancer (GC) was the primary cause of Krukenberg tumors, with colorectal cancer (CRC), appendix mucinous adenocarcinoma, and breast, prostate, and cervical cancer. It has been proposed that the incidence of CRC associated with the Krukenberg tumor has progressively increased in recent years, surpassing that of the giant cell tumor (GCT) with the Krukenberg tumor [5]. Before, any ovarian cancer that spread to other areas was called a Krukenberg tumor. To avoid any confusion, Novak and Grey developed new diagnostic criteria. Consequently, a Krukenberg tumor is defined as a signet ring cell cancer in the thick fibroblastic stroma of the ovary that secretes mucin [6].

## Case presentation

**Figure 1:**
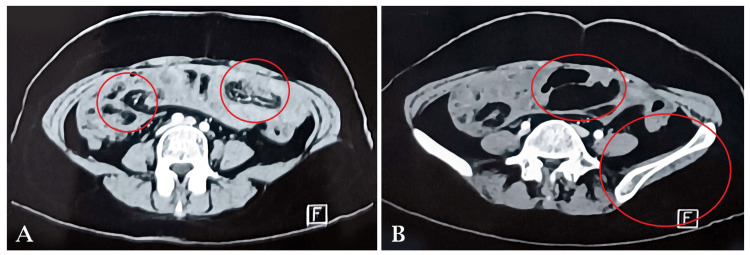
A) Mild diffuse thickening of the omentum. B) Moderate volume of free fluid in the abdomen and pelvis with diffuse peritoneal thickening.

We present the case of a 34-year-old female admitted to the hospital with a complaint of pain in the abdomen. The patient was alright 15 days back then she started developing pain in the abdomen, which was in onset and gradually progressive not radiating to any part of the body. The abdominal examination is previously done. The previous computed tomography (CT) scan of the abdomen shows (Figure 1A) mild diffuse thickening of the omentum and (Figure 1B) free fluid in the abdomen and pelvis with diffuse peritoneal thickening.

Mild omental thickening and soft tissue nodularity are seen along the upper abdomen and gross ascites are seen. This is a known case of carcinoma of the ovary and reported history of stage 3c 32 cycle chemo received. The patient's general examination is normal, while the systemic examination shows the central nervous system (CNS) and cardiovascular systems as alright. The patient had done all routine examinations. According to reports of general hematology, all results were normal.

**Figure 2:**
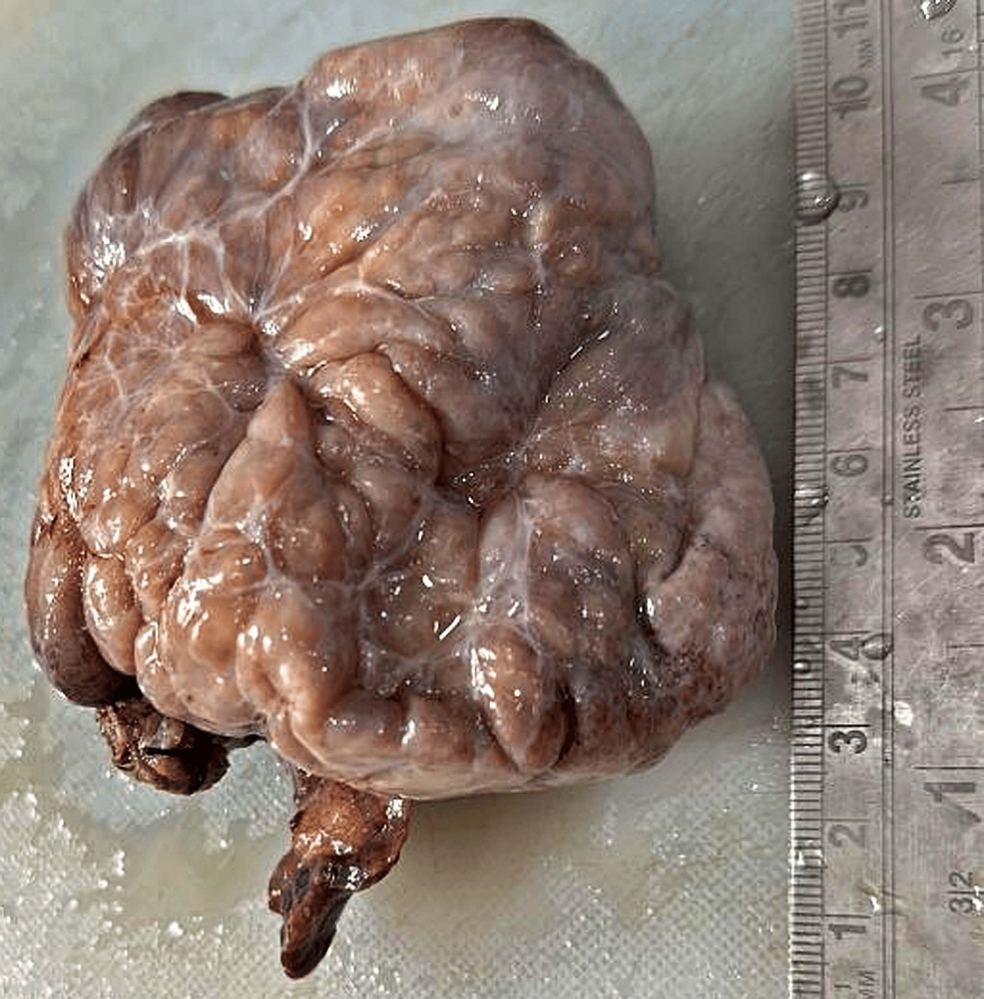
Right ovary with fallopian tube.

The patient underwent surgery under spinal anesthesia, during which a salpingo-oophorectomy was done, under all aseptic precautions. Both the fallopian tubes were removed along with bilateral ovaries and sent for histopathology. For histology examination, the specimen in the two containers received the right ovary measured 11×9×3.5 cm, with the fallopian tube measuring 3.5 cm in length. The grossly external surface of the right ovary is lobulated and soft (Figure 2).

**Figure 3:**
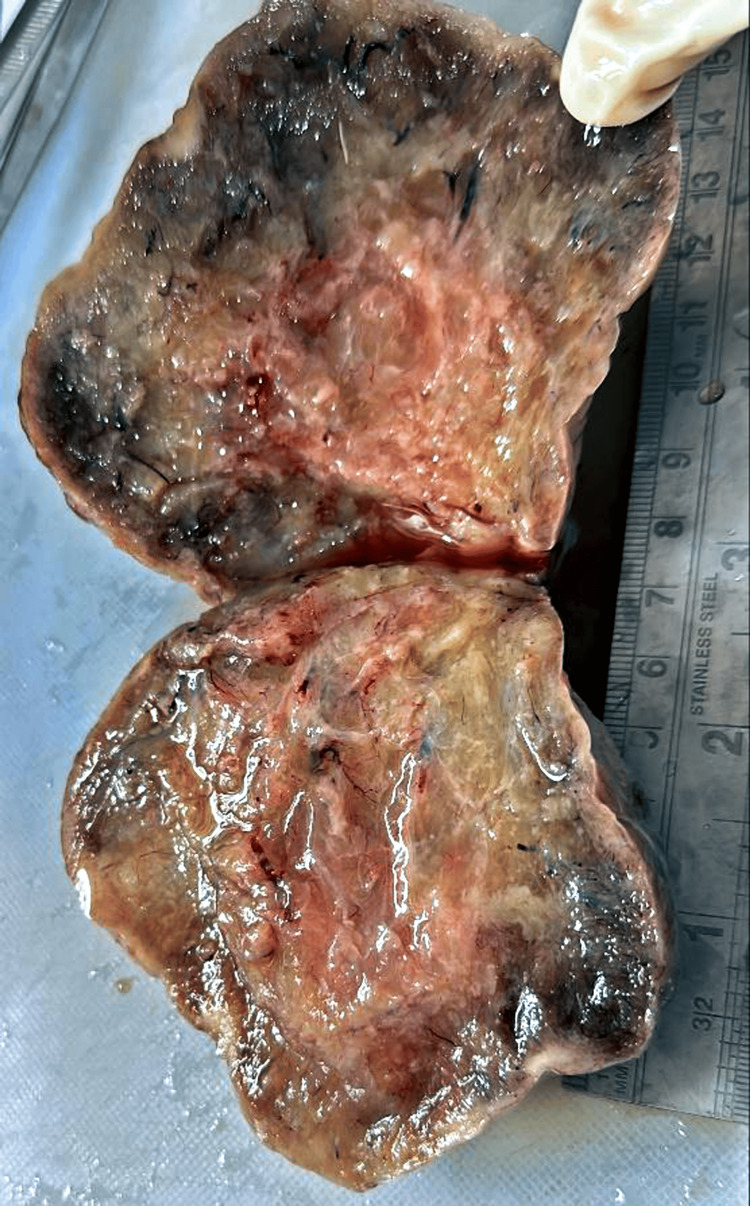
The right ovary was observed as lobulated with soft areas.

The right ovary was observed as lobulated, and a soft area was attached to the fallopian tube. The whole specimen measured 11×10×3.5 cm (Figure 3).

**Figure 4:**
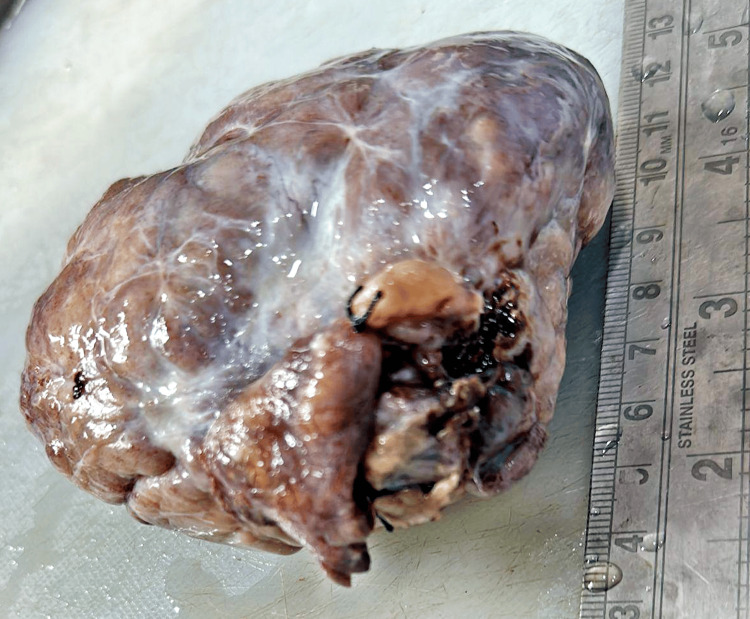
Left ovary attached to the fallopian tube.

Container two is labeled as an excised specimen of the left ovary and received an encapsulated specimen of the left ovary with an attached fallopian tube. The whole specimen measures 7×5.5×3 cm, and the left ovary measures 5.5×4×3 cm (Figure 4).

**Figure 5:**
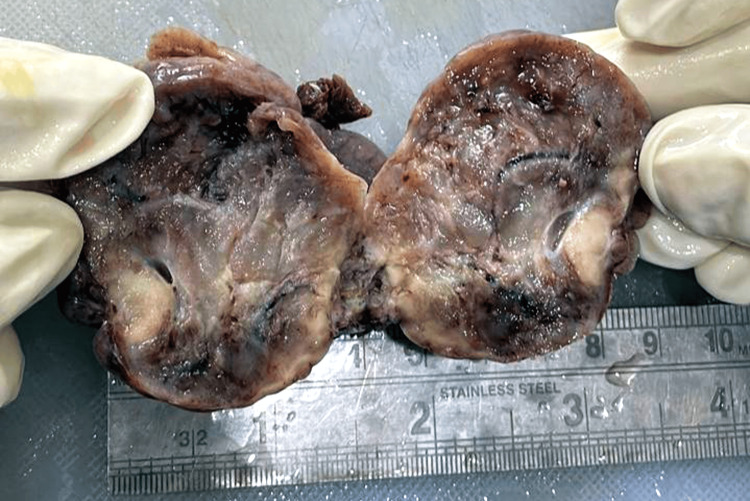
The left ovary's cut section observed a white solid hard area.

The attached left fallopian tube measured 4 cm in length. The external surface of the left ovary was smooth, lobulated, and soft to firm. Solid areas were identified on the cut section, and one greyish-white solid hard area measured 1×1 cm was identified (Figure 5).

**Figure 6:**
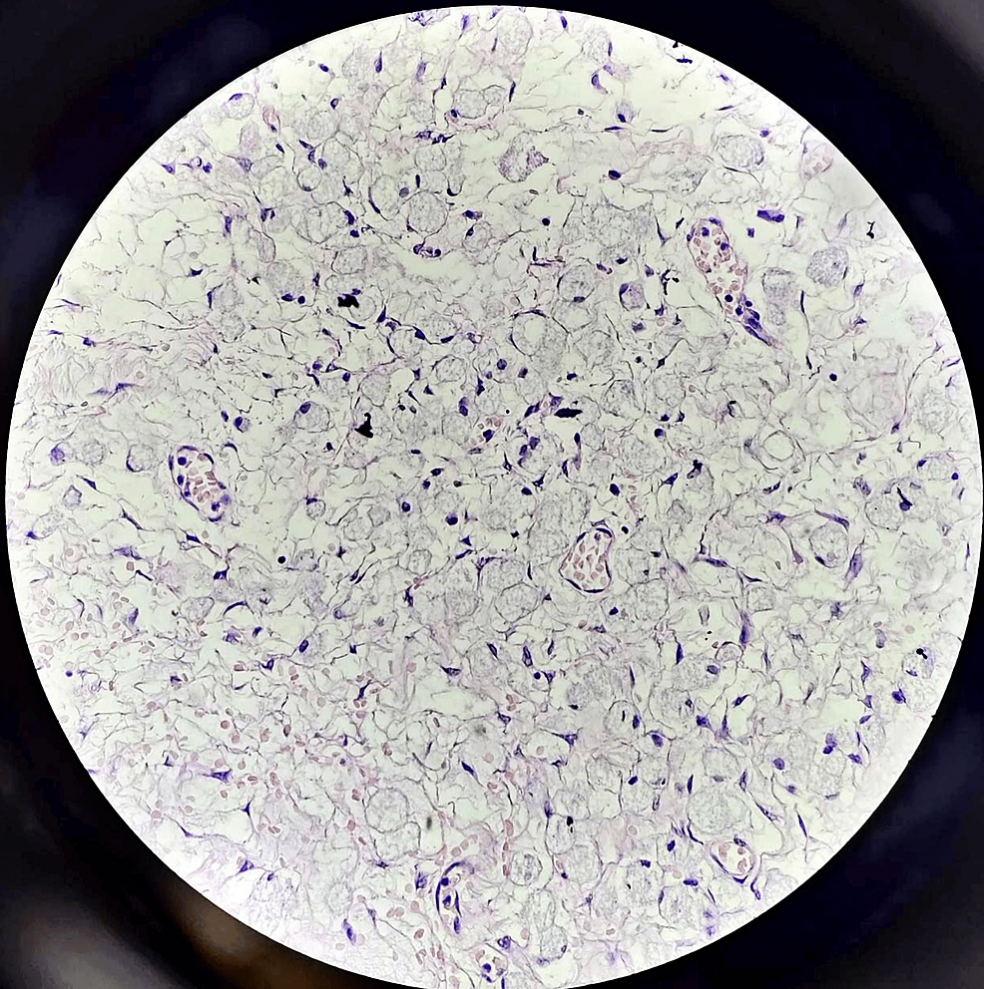
Microscopy showing epithelial tumor with mucin-secreting signet ring cells observed.

Operative histopathology revealed the results of an epithelial tumor, with mucin-secreting signet ring cells and highly suspicious of the Krukenberg tumor (Figure 6).

The right fallopian tube is positive for infiltration by malignant cells. After surgery, the patient was shifted to the ICU for observation, one blood transfusion was done after OT, and then the patient was shifted to the ward. The treatment drugs were given to patients during hospitalization (Table 1). They allowed the patient to have a full diet as recommended by the dietician. The patient was vitally stable, and she was advised for palliative chemotherapy by the surgery department with treatment shown in (Table 2) and was discharged from the hospital. 

**Table 1:** Treatment drugs are given to the patient in hospital. BD: bis in die; TDs: ter die sumendum; OD: once daily

Drugs names	Doses	Duration
Magnet Fort IV BD	1.5 g	7 days
Metro 100 mL IV TDs	1,000 mg	Every 8 hours for 6 days
Pan 40 mg IV OD	40 mg	10 to 15 days
Dynapar AQ IV BD	75 mg	2 days
Tramadol IV 100 TDS	2 mL	Every 4-6 hours for 15 days

**Table 2:** Treatment is given to the patient after discharge. BD: bis in die; TDs: ter die sumendum; OD: once daily

Medicine	Quantity	Doses	Mg	Duration
Protein powder	1	TDS	-	10 days
Tablet paracetamol 650	10	BD	650 MG	10 days
Tablet pantoprazole	10	OD	40 MG	10 days
Tablet chymotrypsin	15	TDS	-	5 days

## Discussion

As per the study by Annal et al., Krukenberg tumors in young patients are extremely uncommon, and 0they should always be considered when diagnosing bilateral solid ovarian neoplasms in patients of any age. In the diagnosis of ovarian tumors, routine use of gastrointestinal endoscopy should be considered [7]. As per the study by Badescu et al., in patients with lung adenocarcinoma, the association between peritoneal carcinomatosis causing urinary tract obstruction and the Krukenberg tumor is infrequent [8]. As per the study by Lin et al., patients with Krukenberg tumors and GC exhibit several clinical characteristics. For Krukenberg tumors originating from the stomach, hypercoagulable conditions, peritoneal metastases, premature chemotherapy, and oophorectomy may be worse indicators [9]. As per the study by Pesce et al., diagnosing ovarian metastases can be clinically and radiologically challenging as they can be signs of original ovarian cancer. Additionally, histological assessment is crucial, and a multidisciplinary approach is necessary to prevent certain mistakes. Indeed, the occurrence of the Krukenberg tumor should be considered in the workup of gallbladder cancer by keeping in mind this rare occurrence in clinical practice [10]. As per the study by An-Chieh Liu et al., the original lesion of a Krukenberg tumor is too tiny to be seen. The primary tumor must be found by careful endoscopic and radiographic examination of the digestive tract. The primary site of the adenocarcinoma can be identified with the help of immunohistochemical analysis [11].

## Conclusions

An uncommon illness that progresses quickly is the Krukenberg tumor. The prognosis of KT, an uncommon illness that advances rapidly, can be improved with the right diagnosis and treatment. In determining the source of metastatic adenocarcinoma, immunohistochemistry staining is helpful. Given their rarity, a national registry should be established to gather data on patients with Krukenberg tumors to enhance treatment outcomes and diagnosis.
